# Nurses’ intention to stay in the work environment in acute healthcare: a systematic review

**DOI:** 10.1177/17449871221080731

**Published:** 2022-07-08

**Authors:** Asma Al Yahyaei, Alistair Hewison, Nikolaos Efstathiou, Debbie Carrick-Sen

**Affiliations:** PhD Student, Lecturer, College of Nursing, Sultan Qaboos University, Sultanate of Oman; School of Nursing, Institute of Clinical Sciences, College of Medical and Dental Sciences, 1724 University of Birmingham, Birmingham, UK; Reader, Institute of Clinical Sciences, College of Medical and Dental Sciences, 1724 University of Birmingham, Birmingham, UK; Lecturer/Adjunct Professor, University of Ottawa, MRes Clinical Health Research Programme Lead, School of Nursing, Institute of Clinical Sciences, College of Medical and Dental Sciences, 1724 University of Birmingham, Birmingham, UK; Foundation Clinical Professor of Nursing and Midwifery Research, School of Nursing, Institute of Clinical Sciences, College of Medical and Dental Sciences, 1724 University of Birmingham, Birmingham, UK

**Keywords:** intention to stay, leadership, nurses, systematic review, work environment

## Abstract

**Background:**

With staffing shortages affecting increasing numbers of health services globally, and predictions that shortages will worsen in the future, there is broad consensus that leaders at all levels must do more to support and develop current employees. However, the wide range of attributes of a healthy work environment identified in the literature and the financial implications of creating healthy work environments make it challenging to determine which elements of the nursing work environment are the most important in terms of workforce sustainability. This is a significant gap in our knowledge, and there is no consensus in the literature regarding definition and explanation of work environment factors in a way that facilitates prioritisation.

**Objectives:**

The aim of this review was to synthesise and evaluate the evidence of the factors which may have an effect on intention to stay and role of the work environment in enhancing nurses’ intention to stay in the work environment in acute healthcare.

**Design and methods:**

This systematic review followed the Preferred Reporting Items for Systematic Reviews and Meta-Analyses statement guidelines. A comprehensive search was performed for relevant articles published between 1990 and December 2017 using the following electronic databases: Allied and Complementary Medicine Database (AMED), Excerpta Medica dataBASE (EMBASE), ProQuest Nursing & Allied Health Source, ProQuest theses and dissertations, Cumulative Index to Nursing and Allied Health Literature (CINAHL) Plus, MEDLINE (Ovid) and PsycINFO. The reviewers independently screened the abstracts and full texts, extracted data, and assessed the methodological quality of the included papers using appropriate tools.

**Results:**

A total of 4968 studies were screened by title, abstract, and full-text review, and 29 studies were included in this review. The identified determinants of nurses’ intention to stay were grouped into four main categories: individual indicators (personal and professional), organisation/profile, work environment, and patient-related. Several working environment variables identified in this review were significantly associated with the nurses’ intention to stay.

**Conclusion:**

Despite the limitations of this review, the evidence indicates that attention to meso-level variables such as organisational characteristics and work environment is vital if the working environment is to improve and nurses’ intention to stay is to increase. The multifaceted nature of the concept of intention to stay makes it difficult to present definitive conclusions based on the findings of this review. However, the identified theoretical models were instrumental in differentiating intention to stay from other concepts such as intention to leave, turnover and retention, theoretically, and operationally.

## Introduction

The World Health Organization (WHO) predicts there will be a shortage of 12.9 million workers in the global healthcare workforce by 2035 (Truth, 2013). The current and growing global nursing shortage is one of the most serious healthcare challenges that various countries are facing (Marć et al., 2019; Oulton, 2006). Concerns about nursing staff were first reported in the United States of America (USA) between 1930 and 1950, when most hospitals increased bed numbers based on the population’s health needs (Murray, 2002; West et al., 2007). However, the global nursing shortage was identified as an acute issue in the early 1990s (West et al., 2007). The current nursing shortage is the result of a complex mix of factors. For example, in the USA, the implementation of managed care, defined as a system of healthcare that emphasises preventative medicine and home treatment, in the 1990s led to significant cuts in nurse staffing, increases in the number of patients within a nurses’ caseload, and a near-freeze in average wages (Murray, 2002; Oulton, 2006).

Although the factors contributing to global nursing shortages vary due to the uniqueness of each country and its healthcare system, there are some common key factors. These include the growing number of retirees due to an aging nursing workforce, an inadequate supply of new nursing graduates, and unfavorable work environments. These factors have led to high staff turnover rates and an uneven distribution of the workforce (Eley et al., 2010; Oulton, 2006). The multifactorial nature of the nursing shortage indicates that its solution requires a combination of approaches to reduce turnover and enhance retention (Hayes et al., 2006; Kleinman, 2004).

Traditionally, research has focused on staff turnover and retention; however, more recently, the focus has shifted to nurses’ intention to leave or stay with their current organisation. In recent years, the intention of nurses to leave or stay in their current organisations and the main predictors of such decisions have become the focus of research, rather than turnover and retention (Jiang et al., 2017; Wang et al., 2018). However, the terms, intention to stay, intent to leave, turnover, and retention are often used interchangeably in the literature, making it difficult to determine the relative impact of different factors. Despite this inconsistency in the use of the terms, it is important to examine potential predictors of each concept and to understand their impact on workforce stability (Buchan, 2010). Intention to leave (ITL) among nurses, as a predictor of nursing turnover, has featured in the literature since the early 1980s, whereas intention to stay (ITS) is a new term (Buchan, 2010; McCarthy et al., 2007). Furthermore, the predictors of ITS often contradict those of ITL. Predictors may include: job characteristics, organisation climate, working conditions, and perceived role value (Gershon et al., 2007).

Generally, there is an assumption that the two concepts (ITS and ITL) have an inverse correlation, where if one increases it results in the reduction of the other. Although the distinction between the two concepts has not been extensively explored in the literature, some researchers have sought to address the question of whether intention to stay and intention to leave are in fact two sides of the same coin (Cho et al., 2009; Howe et al., 2012; Nancarrow et al., 2014). In general, they conclude that despite the terms sharing a number of similarities and some overlap in relation to predictors of intention to leave and intention to stay, the two concepts are not simply opposing terms (Nancarrow et al., 2014; Ghosh et al., 2013; Cho et al., 2009). Intention to stay was chosen as the phenomenon of interest for several reasons. First, intention to stay has been postulated as the single best early negative predictor of actual turnover (Cho et al., 2009; Tourangeau et al., 2010), and so investigating it will yield a proactive, modifiable indicator rather than waiting for the actual turnover to take place. Second, the focus on intention to leave has resulted in intention to stay being overlooked in nursing research; thus, this is the first systematic review which examined nurses’ intention to stay’s factors. It is proposed that intention to stay is a multistage process, even though it may seem (by some) to be a passive decision when compared to intention to leave (Tourangeau et al., 2010).

Working conditions and the work environment are predictors that have been explored in greater depth (AbuAlRub et al., 2016; Alhamwan and Mat, 2015; HanTrinkoff and Gurses, 2015; McCarthyTyrrell and Lehane, 2007). In light of this evidence, healthcare organisations around the world have identified a need to develop, maintain and improve the quality of the work environment to promote the recruitment and maintain the retention of high-quality staff. Thus, the need for a healthy work environment has focused attention on the impact of this concept on workforce stability.

However, gaps remain as there is no consensus in the literature regarding the definition and priority of factors that contribute to a healthy work environment. A systematic review is needed to identify and evaluate the literature pertaining to the nursing work environment and explore the direct and indirect impact on nurses’ decision to stay with their organisation. The primary focus of this review was to examine if there was a relationship between a healthy work environment and ITS. Furthermore, the review aimed to identify, synthesise and evaluate the evidence regarding the impact of the work environment in relation to ITS of nurses in the acute healthcare setting.

## Methods

### Search strategy, data sources, and screening

The healthcare systematic review guidelines of the Centre for Reviews and Dissemination (CRD) and the Preferred Reporting Items for Systematic Reviews and Meta-Analyses (PRISMA) guidelines were followed to develop a systematic and rigorous review (CRD, 2009; Moher et al., 2009). An initial limited search through PROSPERO, COCHRANE, and MEDLINE databases was undertaken using the keywords: nurses, work environment, workplace, and ITS. The result of this initial search was used in the formation of the review question, development of search terms (Table 1), and the systematic review protocol. The Population, Intervention Comparison, and Outcome (PICO) model are commonly used in systematic reviews. However, a modified PICO model was used to develop the review question and develop the search terms (P: population, I: indicator, C: context, O: outcome) (Pollock and Berge, 2018; Walker, 2014). This systematic review’s question was constructed a in non-interventional context; thus, the modified format of PICO was utilised. Based on the initial search and Librarian consultation, the following databases were searched: AMED (EBSCO), EMBASE, ProQuest Nursing & Allied Health Source, ProQuest theses and dissertations, CINAHL Plus (EBSCO), MEDLINE (Ovid), and PsycINFO. The search period was between January 1990 and December 2017.

**Table 1.** Search terms based on PICO.

	Question component and search terms	Type of terms		Boolean operator
		Free	Mesh	
Population	Nurses	Nurse, nursing	Nurs*	Or
		Nursing personnel		
				And
Indicator	Work environment	Work environment		Or
		Workplace	Workplace, work site	Or
		Work setting		Or
				And
Context	Acute healthcare setting	Healthcare setting		Or
		Healthcare facility		Or
		Healthcare organization		Or
		hospital	Hospital	Or
				And
Outcome	Intention to stay	Intent to stay, remain		Or
		Intent to leave, quit, resign		Or
		Turnover, retention		Or

### Inclusion and exclusion criteria

Articles were included in the review if they reported quantitative studies that examined acute-care nurses’ ITS in their organisation, were written in English language and published between 1990 and 2017. The review included studies of qualified nurses of any grade/level including Registered Nurses (RNs), Licensed Practical Nurses (LPNs), Registered Practical Nurses and Licensed Vocational Nurses (LVNs). Qualitative studies, abstracts, review articles, and studies examining ITL were excluded (Table 2). Studies of mixed staff populations, or those that used a mixed-methods design were included if quantitative data relating to nurses was clearly accessible in the findings. With limitations in number of studies which were conducted outside the acute care setting, the researcher decided to focus on nurses working in acute healthcare settings in this systematic review.

**Table 2.** Inclusion and exclusion criteria.

Inclusion criteria	Exclusion criteria
Published quantitative studies, including quantitative data from mixed-methods studies, were included provided that the quantitative aspect of the study was clearly reported	Qualitative studies and grey literature (except theses and dissertations)
Studies containing empirical data (primary or secondary)	Non-empirical articles (including literature reviews [including systematic reviews]), discussion articles, commentaries, short communications, expert opinion articles, and editorials and letters to the editor
Studies examined the intent to stay (or equivalent term such as intention to remain), work environment (modifiable and functional elements of work environment)	Studies examined other concept such as intention to leave, turnover and retention
Written in English language	Written in non-English language
Examined any grade/level of nursing who provided direct patient care including registered nurses (RNs), licensed practical nurses (LPNs), registered practical nurses and licensed vocational nurses (LVNs)	Examined non-nursing populations or nurses who did not provide direct patient care
Published after 1990	Published before 1990

### Screening

**Figure 1.**
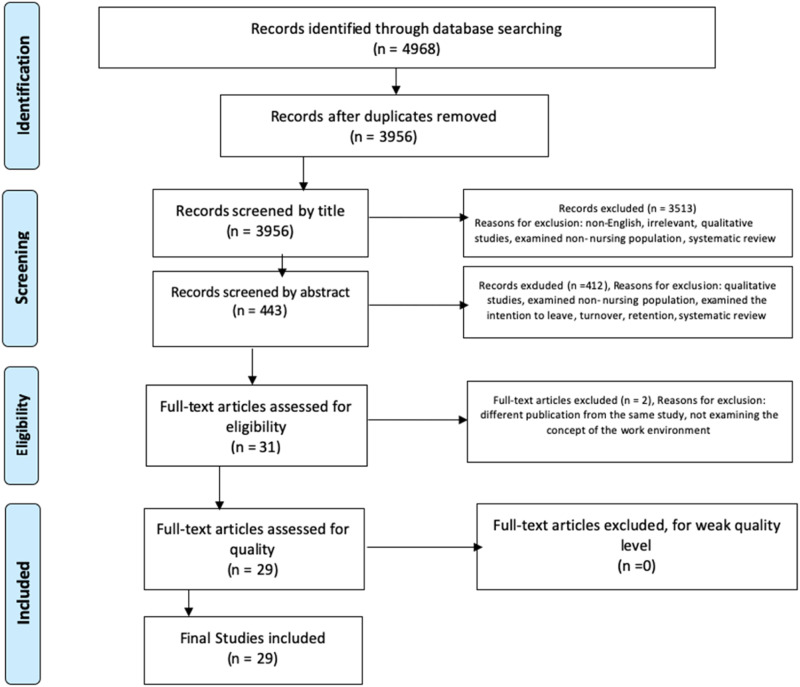
Preferred Reporting Items for Systematic Reviews and Meta-Analyses (PRISMA).

Database searches were completed by one reviewer (first author), and the results from each database were entered into a reference management software package (EndNote v8). Duplicate articles were removed, and the titles of the remaining articles were screened by one reviewer (first author). Studies not related to the topic were removed. The abstracts of the remaining articles were screened by two reviewers (first author and fourth author) independently and were retained, if they met the inclusion criteria. Discrepancies were resolved through discussion. A total of 29 articles met the criteria for inclusion, based on full-text retrievals (Table 2 and Figure 1). All selected articles were reviewed by two members of the team to verify eligibility. It was agreed a priori that should more than one paper report the same study, then the most recent publication would be accepted. All selected 29 articles reported different studies.

### Data extraction

Since all the retrieved studies were non-interventional studies, data from the final included articles were extracted using a modified version of the “data collection form for intervention review — randomised controlled trials (RCTs) and non-RCTs” developed by the Cochrane Collaboration (Cochrane, 2014). The modified version has been used in reviews to extract data from quantitative non-interventional studies. However, to ensure the information to be extracted was standardised and relevant, the data extraction form was pilot-tested and discussed prior to use by two reviewers (first author and fourth author). No changes to the data extraction form were needed. The following information was collated: author, publication year, journal, country, research purpose or objective, design, theoretical framework, sample and population, assessment tools, reliability and validity, response rate, statistical analysis, results, limitations, and the quality assessment result. This information was compiled in a summary table (Supplementary Table S1). To minimise data extraction errors and maintain consistency, data from each paper were extracted by two reviewers independently, one reviewer (first author) extracted data from all selected articles, and the second data extractor was one of the other review team members (second author, third author, and fourth author).

### Quality appraisal

We modified the Appraisal Tool for Cross-Sectional Studies (AXIS tool) (Downes et al., 2016) to assess the quality of the selected articles. The original AXIS tool does not include a numerical scale that can be used to produce a quality assessment score because it is designed to assess the individual characteristics of a study cumulatively. The appraisal form has sections in which a “Yes,” “No,” or “Do not know” response to each question can be recorded. All answers can be supplemented with additional comments. However, in the modified version, the answer “Do not know” was replaced with the option “Unclear,” and a fourth option of “Partially” was added to address inadequate reporting of studies’ details. The response options were assigned a numerical score as follows: Yes, 3; Partially, 2; Unclear, 1; and No, 0. There were 17 appraisal questions, and based on the cumulative score, studies were divided into three categories: poor quality (0–16), moderate quality (17–33), and high quality (34–51) (form available in the Supplementary Material). Two reviewers conducted the appraisals independently and when there was a divergence (*n* = 2) in scoring, the reviewers discussed the item until consensus was reached (Downes et al., 2016). The overall purpose of the quality assessment was to ensure that moderate- and high-quality studies (17–51) were included and poor-quality studies (0–16) were excluded.

### Data synthesis

Narrative synthesis was undertaken to summarise and analyse the studies’ findings (Ryan and Consumers, 2013). This was necessary because the heterogeneity of the studies’ populations, assessment tools, main concept definitions and outcome measurements, precluded merging the results, and undertaking a meta-analysis (Popay et al., 2017; Ryan and Consumers, 2013). A preliminary synthesis of the studies’ findings was achieved by data tabulation, which has been recommended as a logical starting point for this type of synthesis (Popay et al., 2017). Based on Popay et al. and following the process of narrative synthesis, the included studies were organised into smaller groups and clusters to make the process more manageable. ITS’s indicators with some similarities were retrieved and were clustered and then were sorted in main groups which included work environment factors, individual indicators (personal and professional), organisation profile and patient-related factors. The characteristics of the individual studies are summarised in a table (Supplementary Table S1).

## Results

### Design

Of the 29 studies that met the inclusion criteria, two employed a mixed-method (quantitative and qualitative) design; however, only the quantitative components were included in this systematic review (Cowden and Cummings, 2015; Yarbrough et al., 2017). The remaining 27 articles, including five PhDs used cross-sectional designs to obtain primary data, and two reported secondary analysis of a data set (Abernathy, 2007; Lacey et al., 2007).

### Temporal and geographical details

The 29 studies can be divided into two distinct time periods. Ten were performed between 2000 and 2010, and 19 were conducted between 2011 and 2017. More than half of the studies were performed in North America (11 in the USA and 4 in Canada). The geographical distribution of the remainder of the studies was as follows: seven in Asia, one in Africa (Ghana), one in Europe (Switzerland), and five in the Middle East (four in Jordan and one in Lebanon) (Supplementary Table S1).

### Settings

All the included studies were performed in acute healthcare settings comprising public, private, teaching, non-teaching, community, and Magnet and non-Magnet recognition hospitals in urban, suburban, or rural areas. Most of the studies recruited participants directly through their employing institutions. However, recruitment in four studies was through a state board or specialty association (Brewer et al., 2016; Nedd, 2006; Riegal, 2012; Ross, 2016). Moreover, with the exception of two studies that were conducted in a single healthcare site (Sourdif, 2004; Yarbrough et al., 2017), the remainder were undertaken in multiple hospital centres.

### Population

The total sample population of the 29 studies was 26,295 qualified nurses. Twenty-five studies included only Registered Nurses (RNs). One study included nurses (37%) and other healthcare professionals (Gilles et al., 2014); however, only the findings relating to nurses were included in the review. Three studies involved both RNs and Practical RNs (PRNs) (Cowden and Cummings, 2015; Owens, 2011; Tourangeau and Cranley, 2006b). However, total PRNs included was around 5% of the total sample population (1446 PRNs). Regardless of variations between countries in defining the scope of practice of RNs, it is known that an RN is a nurse who has fulfilled the requirement of the nursing program at university or college level and successfully receives a license/certificate/qualification to practice nursing, whereas a PRN is still in training, working under the supervision of the RN.

### Summary of quality review

All 29 studies were rated as moderate- or high-quality studies (Supplementary Table S1). Most of the studies included a justification of the sampling technique and sample size, maintained participants’ anonymity, obtained ethical approval, assured the measures’ reliability, and used theory to guide the study. On the other hand, the most frequent limitations of the studies included the use of a cross-sectional design, reliance on self-reported data, lack of a random sample, low response rates, lack of evidence related to validity assessments for the measure/s used, and lack of detail about the management of missing data and outliers. Eight studies reported response rates lower than 50%, and five studies did not report a response rate. All the studies reported how participant anonymity was maintained and that ethical approval had been obtained, except for the studies by Gilles et al. (2014) and Nakamura et al. (2010). Fourteen studies did not report whether participants’ informed consent was obtained.

### Theoretical frameworks

Almost 60% of the studies (17/29) reported the development or use of a theoretical framework, which guided the research or explained the findings. Six different frameworks (Table 3) were used, which focused on different work environment aspects and their relationship with ITS. In addition, leadership/management theories featured in four studies (Asamani et al., 2016; Cram, 2013; Manning, 2014; Nakamura et al., 2010).

**Table 3.** Theoretical/conceptual framework.

Theoretical framework	Used by
Organizational Dynamics Paradigm of Nurse Retention (Taunton et al., 1997)	(Sourdif, 2004)
Expanded Price Model of Turnover (Price, 2001)	(Brewer et al., 2016)
Cowden and Cummings' Theoretical Model of Clinical Nurses’ ITS (Cowden and Cummings, 2012)	(Cowden and Cummings, 2015; Liang et al., 2016)
Conceptual Model of Intent to Stay (CMIS), which was adopted from (Boyle et al., 1999)	(Gilles et al., 2014)
Conceptual Model of Behavioral Intentions (CMBI), which was developed based on an integrated causal model of turnover behavior (Mueller and Price, 1990)	(Gregory et al., 2007)
Fishbein and Ajzen’s Expectancy–Value Theory (Fishbein and Ajzen, 1974)	(Yarbrough et al., 2017)
Determinants of nurse intention to Remain employed based on Conceptual Model of Intent to Stay (Boyle et al., 1999)	(Tourangeau & Cranley, 2006b; Wang et al., 2012)

### Measurement of independent and dependent variables

The tool used for assessing the different elements of the work environment in five studies was the Revised Nurse Working Index (NWI-R). The Individual Workload Perception Scale-Revised was used in three studies, the Practice Environment Scale of the Nursing Work Index (PES-NWI) was used in two studies and the Farley’s Nursing Practice Environment Scale was used in one study. The tools used had been validated in earlier studies, however, a number of studies reconfirmed the validity and reliability of tools used (Supplementary Table S1).

The outcome of ITS was measured in all studies; however, some used an existing validated tool, whereas others used a new one developed for the purpose of the study. Ten studies used single-item measures to assess ITS. One study asked participants how many years they were planning to stay (Yarbrough et al., 2017); the remainder of the studies used either dichotomous or continuous measures. Four studies used a single dichotomous measure to assess nurses’ ITS, and the result was reported as the percentage of nurses who planned to stay.

Six studies used three to six items to measure ITS. Other instruments used in the studies included the four-item Nurses’ ITS scale developed by Taunton et al. (1997) (one study), the four-item ITS scale developed by Kim et al. (1996) (one study), the Chinese version of the Nurses’ ITS scale created by Tao and Wang (2010) (two studies), and the McCain Behavioral Commitment Scale (McCloskey, 1990) (three studies). The validity of these measures has been previously established in the literature (Kim et al., 1996; McCloskey, 1990; Tao and Wang, 2010; Taunton et al., 1997). Cronbach’s alpha of the ITS scales ranged from 0.695 (Asamani et al., 2016) to 0.91 (Sourdif, 2004), which indicated acceptable to excellent levels of internal consistency (Taber, 2018).

### Intention to stay results

Overall, the included studies reported a wide variation in nurses’ ITS with their organisation. Of the studies using a dichotomous item to assess the percentage of nurses who planned to stay, 95% of nurse respondents working in acute care and ICUs in one Magnet hospital and one non-Magnet hospital in the southwest United States reported their ITS with their current employers (Cram, 2013). The percentage was lower (60% of respondents) in two other US-based studies (Letvak and Buck, 2008; Riegal, 2012). When considering the geographical regions, studies conducted in the USA reported the highest level of ITS. However, USA nurses’ ITS mean score varied depending on work settings, such as whether the nurses worked in suburban (3865) or urban (3731) areas, or in a Magnet hospital (3.92), aspiring Magnet hospital (3.72), and/or a non-Magnet hospital (3.64).

In Canada, Sourdif (2004) found that 50.4% of RNs working in a 400-bed university hospital in Montreal reported their ITS in their current job, whereas only 29.0% reported their ITS in their current hospital. In the three studies that used the McCain five points scale to assess the nurses’ ITS in Jordan, the mean score ranged from 2.83 to 3.12 which indicated a moderate willingness to stay. However, a significant difference was identified between nurses in Jordan working in teaching and non-teaching hospitals where nurses in non-teaching hospitals intended to stay at their current jobs (mean = 3.22, SD = 0.81) longer than nurses in teaching hospitals (mean = 2.86, SD = 0.80; *p* =0.01) (Al-Hamdan et al., 2017; Mrayyan, 2007; Mrayyan, 2008). In China, two studies were conducted 5 years apart examining ITS among nurses working in different general hospitals. Both studies used the ITS five-point scale developed by Wang [38] and reported mean scores of 3.85 (SD 0.82) and 3.53 (SD 0.64), respectively, reflecting the nurses’ strong willingness to stay working as a nurse (Wang et al., 2018; Wang et al., 2012).

### Variables associated with intention to stay

Thirty-one factors were identified in the studies as having a positive or negative impact on the nurses’ ITS in their current position, or with their current employer, or in nursing as a lifetime career. The ITS indicators identified in the studies were synthesised using narrative synthesis into the following main categories: work environment factors, individual indicators (personal and professional), organisation profile, and patient-related factors.

### Work environment factors

Regardless of the impact of other categories on the nurses’ ITS, most attention in the studies was given to the impact of the work environment and working conditions. The purpose of this review was to assess the relationship between work environment attributes and nurses’ ITS. The work environment factors identified were further defined as modifiable and functional variables within the nursing workplace. They related to the work process, experience, and interaction with surroundings.

### Overall work environment

To assess the concept of the work environment, AbuAlRub et al. (2016) and Al-Hamdan et al. (2017) examined the association between the work environment and nurses’ ITS using two different versions of the Nurse Work Index scale. These studies focused on the extent to which nurses control their environment, receive support from their organisation and leadership, as well as the notion of good nurse–physician relationships. These were all identified attributes of a supportive nursing work environment. The results of the logistic regression analysis by AbuAlRub et al. (2016) indicate that receiving accommodation from employers, nurses’ job satisfaction and overall work environment were predictive variables for level of ITS. Al-Hamdan et al. (2017) found that the nursing work environment was positively associated with nurses’ ITS (t = 4.83, *p* <.001), and the ITS score increased by 3.6 points for every one-unit increase in the total PES-NWI score on average.

### Teamwork interactions

Professional interactions, factors such as teamwork, workgroup cohesion, collaboration, and co-workers/peer support were also identified as predictors of nurses’ ITS (Abernathy, 2007; Kaewboonchoo et al., 2014; Tourangeau and Cranley, 2006b). However, the impact of peer support on ITS was not constant in the study by Abernathy (2007), in which two regression models were tested (linear and logistic). Peer support was a significant positive predictor in the linear regression only when ITS was measured with a continuous scale. However, this significance was not detected when ITS was converted to a dichotomous scale in a logistic model.

### Leadership practices/nursing empowerment

Nine studies investigated the influence of nurse managers’ leadership practices, behaviour and styles on several nursing outcomes, mainly in association with workforce stability (Asamani et al., 2016; Brewer et al., 2016; Cowden and Cummings, 2015; Cram, 2013; Liang et al., 2016; Manning, 2014; Nakamura et al., 2010; Sourdif, 2004; Wang et al., 2017). Although there were methodological differences in the studies, there was some agreement on the effect of leadership on nurses’ ITS. However, different leadership styles have been studied extensively to identify the best style to increase workforce stability and nurses’ ITS. Asamani et al., (2016) found a weak but significant positive correlation between supportive (r = 0.221), participative (r = 0.243) and achievement-oriented leadership styles (r = 0.184) and staff ITS. In Asamani et al.’s prediction model, nurse managers’ leadership styles were responsible for 13.3% of ITS of nurses in their current job position. Similarly, Mrayyan (2008) identified the managers’ decision-making styles as predictors of the nurses’ ITS. Although transformational leadership is advocated in the nursing literature as a desirable leadership style that positively influences nursing workforce stability, other styles including transactional and passive-avoidant leadership styles failed to predict nurses’ ITS in a regression analysis (Manning, 2014). This may highlight the need to focus on leadership practices that enhance nurses’ decision to stay more than adapting a specific or single leadership style.

One study investigated the impact of Management by Objective (MBO) and used a stepwise multiple logistic regression analysis of 44 evaluation items for MBO. Five items were recognised as significant independent variables of ITS: appreciating the hospital’s atmosphere, maximum utilisation of their abilities, feeling an attachment to one’s current job, and consciousness of personal objectives and motivation (Nakamura et al., 2010). However, the study of Nakamura and colleagues reflected an attempt to construct a path network that could sufficiently explain the intention to remain employed. Furthermore, using one leadership style was found to partially explain the increased intention of staff nurses’ to remain employed in their current position. In addition to leadership practices, nursing empowerment was examined in two studies and was found to be a significant predictor of nurses’ ITS (Cowden and Cummings, 2015; Nedd, 2006). In the seminal work of Cowden and Cummings, empowerment, organisational commitment and desire to stay explained 63% of the variance in ITS. Moreover, empowerment was found to mediate the influence of leadership practice on nurses’ ITS (Cowden and Cummings, 2015). This suggests that the causal influence of leadership practice as an independent variable on the development of nurses’ behavioural intention to remain in their current position was indirect and its impact was mediated by nursing empowerment. In other words, leadership practice had a positive impact on enhancing the nursing empowerment and directly increasing the nurses’ willingness to stay in their current position; however, the precise mechanism at work was not clear.

### Organisational support and climate

Nurses’ perception of nursing organisational support predicted 30% of the variance of nurses’ choices to work again for their current employing organisation (Riegal, 2012). Structural equation modeling analysis by Gregory et al. (2007) provides partial support for the impact of organisational culture, trust and satisfaction on ITS, accounting for 31% of the variance. Furthermore, both Mrayyan (2008) and Ya-Ting (2017) examined the influence of the hospital organisational climate on nurses’ ITS and concluded there was a significant positive correlation between hospital organisation climate rating and nurses’ ITS.

The hospital organisation climate, along with other identified predictors, explained 12% and 50.8% of the variance of nurses’ ITS, respectively (Mrayyan, 2008; Ya-Ting, 2017). Although Ya-Ting (2017) did not provide a definition of organisation climate, Mrayyan (2008) defined it as the reported perceptions of nurses about the organisation, administrative support, quality of care, nursing leadership, and professionalism. However, the lack of explanation of the individual influences of these items on the nurses’ ITS is a limitation of the study. Another variable is nurses’ perceptions of the safety climate, which has been identified as a predictor of nurses’ ITS and as a mediator of the indirect influence of transformational leadership on the nurses’ willingness to remain employed (Liang et al., 2016).

### Adequate resources

Nurses’ perception of their workload, adequate staffing and number of working hours per week (Abernathy, 2007; Gilles et al., 2014; Liang et al., 2016), the availability of professional advancement and opportunities for promotion (Brewer et al., 2016; Wang et al., 2012; Yarbrough et al., 2017), and the availability of mentor support (Brewer et al., 2016) influenced ITS. For example, perianesthesia nurses who worked in different healthcare settings in the USA who had an assigned preceptor had a statistically significantly higher ITS score (4.12) compared to those with no assigned preceptor (3.97) (Wang et al., 2012).

### Individual factors

Twenty-one studies identified a total of 16 individual factors influencing ITS. These included personal characteristics, such as age, level of education, and professional characteristics including position and professional experience. Age and job satisfaction were the most frequently identified strong predictors of nurses’ ITS. Two subsets of factors were considered among individual determinants: sociodemographic characteristics and psychological cognitive experiences.

In terms of sociodemographic characteristics, 10 studies concluded that older nurses were more likely to stay in their current hospital than younger nurses. In fact, Owens (2011) who examined nurses’ ITS beside other nursing outcomes such as job satisfaction and organisation commitment, concluded that differences between generational cohorts were associated significantly with the nurses’ ITS. Owens (2011) compared ITS among three nursing generations. The one-way analysis of variance (ANOVA) conducted was significant [F (2, 313) = 4.49, *p* <.05], and the post-hoc analysis showed a significant difference between Boomers (1946–1963) and Millennials (1981–2000), with Boomers having a higher ITS score. Similarly, Liang et al. (2016) concluded that nurses older than 36 years were more likely to stay in their job. However, Nedd (2006) concluded that age, along with other individual characteristics, was not significantly correlated with ITS.

Another time-related factor reported in the studies was years of employment in the current hospital or in the nursing profession. Younger nurses with fewer years of experience were less likely to stay in their current position (Gilles et al., 2014; Liang et al., 2016), although age-related predictor variables based on a structural equation model explained only 9% of the variance (*p* < .001) of nurses’ ITS (Liang et al., 2016).

A regression analysis conducted by El-Jardali et al. (2013) revealed that nurses less likely to report ITS were younger, unmarried, and had fewer years of work experience compared to older married nurses. Furthermore, Sourdif (2004) and Kaewboonchoo et al. (2014) concluded that married nurses were more likely to express their willingness to stay with their current employer. Nurses’ educational level has also been identified as a significant, negative predictor of nurses’ ITS. Several personal characteristics were identified as positive indicators of ITS, such as having a higher position, being certified in a nursing specialty, being a LPN, having a full-time job, and having made a personal choice to work in their current organisation. Additionally, ethnicity/race (being white) and availability of non-local job opportunities for nurses were negative predictors of nurses’ ITS (Brewer et al., 2016).

Job satisfaction was examined in 10 studies and was positively correlated with nurses’ ITS. Job satisfaction was presented in some of the models as nurses’ emotional response to work (Al-Hamdan et al., 2017; Cowden and Cummings, 2015). However, in this review, overall job satisfaction was classified as a personal factor, whereas satisfaction with particular aspects of the work environment was regarded as work environment factors. Organisation commitment accounted for 31% of the variance in the model of clinical nurse ITS developed and tested by Cowden and Cummings 2015. Similarly, Brewer et al. (2016) and Riegal (2012) concluded that organisation commitment had a positive predictive relationship with choosing to work again for the current organisation.

Burnout and emotional labour were negatively associated with nurses’ ITS (Liang et al., 2016; Tourangeau and Cranley, 2006b) although nurses with higher levels of emotional intelligence were more likely to stay in their current position (Wang et al., 2018). The relationship between age and job satisfaction, as independent variables, and nurses’ ITS was one of the most sustainable relationships across the literature. A positive relationship was identified between age and job satisfaction in several studies (Brewer et al., 2016; El-Jardali et al., 2013; Owens, 2011; Tourangeau and Cranley, 2006b).

### Organisation/unit profile factors

Nurses who worked in tertiary hospitals were more likely to report higher ITS scores than those working in secondary or primary level hospitals (Wang et al., 2012). Although (Mrayyan, 2007) concluded that nurses employed in non-teaching hospitals in Jordan were more likely to stay in their position than nurses’ working in teaching hospitals. However, another study conducted in Jordan by Al-Hamdan et al. (2017) found significant differences between the ITS score among nurses who worked in public hospitals relative to teaching hospitals at the same level of nursing work environment score (t = −4.29, *p* <.001) where nurses working in public hospitals were more willing to stay.

In Lebanon, a similar study examined the level of job satisfaction and nurses’ ITS. Its findings revealed that nurses working in primary care in rural areas were more satisfied with all aspects of work compared to their hospital counterparts and had a significantly higher ITS [50]. In Taiwan, nurses who worked in special units (outpatient clinic, emergency room, operating room, dialysis, and psychiatric) had a stronger ITS at their hospitals than those who worked in ICUs (Ya-Ting, 2017).

However, the evidence identifying the significance of the impact of hospital classification on nurses’ ITS was inconsistent. Classification based on Magnet status was the only consistent relationship identified. Nurses working in Magnet hospitals tended to remain employed longer than nurses working in non-Magnet institutions [25,42]. This finding was not surprising considering the strong evidence base that has accumulated over the last two decades that report superior work environments and better nurse outcomes, such as lower levels of nurse dissatisfaction and burnout, which have been achieved by hospitals with Magnet recognition compared to non-Magnet-accredited organisations (Kelly et al., 2012).

### Patient-related factors

Patient-related factors reflected the patient response to their care and included the nurses’ perception of the care provided and praise and recognition received from patients and their families. Only two studies referred to patient-related factors and reported them as strong predictors of the nurses’ ITS in their organisation and profession (Letvak and Buck, 2008; Wang et al., 2012). However, in the literature little attention was given to the impact of these factors on nursing workforce outcomes such as nursing turnover or their ITS or ITL. Unsurprisingly, more attention was given in the literature to understanding the impact of the nursing workforce on patient care and satisfaction.

### Rapid review of recent publications

Given the importance of workforce issues globally, there is an increasing amount of research being undertaken in this area and new articles have been published since the original review was conducted. This review included evidence published between 1990 and 2017. A further search of the databases (AMED (EBSCO), EMBASE, ProQuest Nursing & Allied Health Source, ProQuest theses and dissertations, CINAHL Plus (EBSCO), MEDLINE (Ovid), and PsycINFO) for the period 2018 and 2020 retrieved four articles summarised in Table 4.

**Table 4.** Summary table of the rapid review.

Author(s) year/Country	Design	Sample and population/Setting/Response rate	Theoretical framework	Assessment tool	Reliability	Validity	Statistical analysis	Results
(Suliman and Aljezawi, 2018)	A cross-sectional and descriptive design was used	A sum of 382 out of 500 nurses from three health care sectors in Jordan	Developed based on the literature review	Individual Workload Perception Revised scale (IWPS-R)	The Cronbach’s alpha of the original scale ranged from 0.61 to 0.90	Tool’s validity had been established earlier	Pearson correlation coefficient	Nurses who have good perceptions of support from their manager and peers, and a manageable workload are more likely to stay in their jobs Nurses working in the public hospitals had significantly better perceptions about their work environment than nurses working in private and university hospitals. Older nurses with lower academic qualifications are more likely to be satisfied with their work
(Li *et al.*, 2020)	A cross-sectional multicentre survey	2,352/3,240 clinical nurses from nine tertiary hospitals in eastern, central, and western China	Developed based on the literature	The nurses' Perceived Organizational Support Scale The Chinese version of the Nurses' Job Control Scale The Chinese version of the Nurses' ITS scale	Cronbach’s alpha was 0.95 Cronbach’s alpha of the total scale and each domain was 0.77–0.92 The Cronbach’s alpha was 0.77	Content validity index (S-CVI) was 0.94 Content validity index was 0.97	Structural equation modeling	Job control perceived organisational support and job satisfaction significantly and directly affected nurses' ITS. Job control and perceived organisational support showed indirect effects on ITS, which was mediated by job satisfaction
(Dorigan and Guirardello, 2018)	A correlational study with quantitative approach	465/1516 nurses with active professional registration	Was developed to be tested	Nursing Work Index Revised (NWI-R)	NWI-R: C alpha 0.80	The tools were previously used	Partial least squares path Modeling (PLS-PM)	The dimensions of practice environment (autonomy, control over the environment and nurse-physicians relationships) predicted job satisfaction (R2 = 43%), safety climate (R2 = 42%) and burnout (R2 = 36%), as well as the intention to stay in the job (R2 = 22%) and in the profession (R2 = 17%) The practice environment showed a strong impact on job satisfaction, safety climate and burnout, with a moderate impact on the intention to stay in the institution and in the profession
					SAQ 0.77–0.85	in the literature and their validity had been established		
					MBI 0.68- o.92			
				Safety Attitudes Questionnaire				
				Maslach Burnout Inventory (MBI)				
(Satoh et al., 2018)	A cross-sectional questionnaire survey was conducted at 12 hospitals in the Tohoku and Kanto regions of Japan	Of the 1034 nurses working in those hospitals, 713 returned the questionnaire (response rate: 69.0%)	Was developed based on the previous literature	24 items were developed based on 16 interviews	Cronbach’s α = 0.66–0.87		Exploratory factor analysis	Variables strengthening intention to stay at the current hospital could be grouped into five factors: “Comfortable workplace environment,” “passive motivational factors,” “convenience of hospital location,” “favorable work–life balance,” and “fulfillment in nursing.”
(Ngabonzima et al., 2020)	A descriptive cross-sectional design with a quantitative approach	185/162 full-time nurses and midwives practising in five selected hospitals with a minimum of 6 months of experience working with their current direct managers	The modified Path-Goal Theory Conceptual Framework (House, 1971)	Path-goal leadership tool	Not mentioned	A pre-test of the data collection tools was conducted with 10 nurses and midwives from a different health facility (hospital) for identification and modification of areas of misunderstanding in the research tools	Regression analysis	Nurses and midwives managers were more inclined to the directive leadership style followed by a supportive leadership style, and the participative leadership style. The nurse and midwife’s managerial leadership styles together significantly explained 38, 10 and 23% of the variance in job satisfaction, intention to stay and service provision, respectively.
								Our study revealed significant weak positive relationships between directive and supportive leadership styles with the intention to stay.

The main findings of the rapid update review of the recent literature highlighted that the relationship between the nurses’ ITS and leadership style was the key area of concern. The findings indicate that leadership behaviours and nurses’ perceptions of staff support were positively associated with ITS among Jordanian nurses (Suliman and Aljezawi, 2018). A study conducted in Rwanda, revealed significant weak positive relationships between directive and supportive leadership styles with nurses’ ITS; however, leadership style accounted for only 10% of nurses’ ITS variance. Ngabonzima, Asingizwe and Kouveliotis (2020) found 38% of the variance of nurses’ job satisfaction was explained by the leadership style.

In one paper three components of the nursing practice environment including autonomy, control over the environment and nurse–physicians’ relationship were examined by Dorigan and Guirardello (2018) and the Partial Least Squares Path Modeling (PLS-PM) analysis revealed the moderate impact of these components on the nurses’ ITS in their current institution and in the profession. Although, the elements of the work environment explained 22% and 17% of the variance of nurses’ ITS in their current organisation and in the profession respectively, they explained 43% of the variances of nurses’ job satisfaction, 42% of safety climate and 36% of nurses’ burnout (Dorigan and Guirardello, 2018).

In a two-stage study, Satoh et al. (2018) identified 24 ITS factors. These were developed and based on interviews with 16 nurses, followed by a cross-sectional questionnaire survey conducted in 12 hospitals in the Tohoku and Kanto regions of Japan. The findings revealed that variables strengthening ITS at the current hospital were grouped into five factors which included; comfortable workplace environment, passive motivational factors, convenience of hospital location, favourable work–life balance, and fulfillment in nursing (Satoh et al., 2018). Among Chinese nurses, job control and perceived organisation support showed indirect effects on ITS, which was mediated by job satisfaction (Li et al., 2020). Thus, job satisfaction significantly and directly affected nurses' ITS (Table 4).

Overall, in the more recent literature, several factors, such as leadership behaviour, organisational culture, autonomy, control over the environment, nurse–physicians’ relationships, and organisation support, appeared as significant factors which influence nurses’ ITS. However, the impact of these factors was more significant on the nurses’ satisfaction compared to the ITS. Consistent with the review of the literature published between 1990 and 2017, recent work published in the last 2 years identifies the importance of the impact of leadership on nursing outcomes. The findings of this brief review provide an overall picture about the impact of the leadership practice on the nurses’ ITS.

## Discussion

To the best of our knowledge, this is the first systematic review to identify and synthesise quantitative research concerning the relationship between nurses’ ITS and the work environment. Twenty-nine studies met the inclusion criteria. There was wide variation in the use of key concepts and tools. Clarification of these concepts and development of consensus regarding their meaning and application are required if the relationships between them are to be investigated rigorously. Key findings from the review relate to individual factors, leadership, and organisational support.

### A complex concept

The concept of ITS in terms of its indicators is complex and multifaceted. Thus, mediator variables play an important role in illustrating the indirect impact of some independent variables on nurses’ intentional behavior. In this review, the mediation effect of job satisfaction and organisation commitment was recognised in several studies, in which they were presented as nurses’ affective and cognitive response to their work. The mediation effect facilitates a better understanding of the relationship between independent and dependent variables when the variables appear not to have a direct impact on the outcome (dependent variable). However, for a concept such as ITS, simple correlation or independent predictor analysis is not sufficient to explain the relationship between ITS and its determinants. Therefore, advanced statistical methods including multivariate analyses and structural equation modeling have been used (Brewer et al., 2016; Cowden and Cummings, 2015; Liang et al., 2016; Tourangeau and Cranley, 2006b). These methods were required to clarify the extent to which the hypothesised variables influenced the ITS and to illustrate the mediating influence of these variables. Searching the field for the concept of ITS and work environment was challenging due to lack of clarity concerning the definition of the key terms. However, measures were taken such as preset inclusion criteria and dual review of the abstracts to identify studies focusing on the main concepts and use of narrative synthesis, where the identified ITS factors were organised into smaller groups and clusters to make the process more manageable. Generally, literature seems to lack clear construct delineation and many constructs seem to overlap and their distinction from others becomes ambiguous.

### Generational issues

A significant association between the work environment and nurses’ ITS was evident in the review. However, the review revealed that individual and demographic factors require consideration when planning strategic interventions to improve the work environment to enhance ITS. For example, age-specific interventions could be more effective in enhancing nurses’ ITS, since individual expectations appear to depend on generation (Owens, 2011; Tourangeau and Cranley, 2006b). Through the literature, some attention was given to examine factors which enhance the intention to stay among older nurses such as nurses’ perception of fairness of the human resources’ practices, financial reasons, flexible schedules, and the availability of continuing professional development to help them keep up with a rapidly changing service (Watson et al., 2003; Armstrong-Stassen et al., 2015; Uthaman et al., 2016). The differences between nurses generations in terms of their intention to stay were linked to diversity in attitude and values among different generations of nurses in the workforce (Tourangeau and Cranley, 2006a). Yet, more research is needed to explore the reasons of these differences between nursing generations.

Job satisfaction also affected the nurses’ decisions to remain employed. This review identified the importance of nurses’ personal attitudes toward their organisations (also known as organisational commitment) in their decisions to remain with their employer. This suggests that younger and newly qualified nurses may be less inclined to stay in one organisation for longer periods. A higher ITS has been observed among nurses who were older, married, had greater experience, high job satisfaction, demonstrated organisational commitment, were in more senior positions, were certified in their nursing specialty, were LPN, had a full-time post, and had made a personal choice to work in their current organisation (Liang et al., 2016; Ross, 2016; Tourangeau and Cranley, 2006b; Ya-Ting, 2017). This finding was consistent across different contexts, settings and organisational cultures. The individual employment needs and expectations of newly qualified and younger nurses need to be investigated to understand their ITS.

### The impact of leadership

A consistent finding in all articles included in the review related to the direct or indirect impact of leadership practice on nurses’ ITS. Different leadership styles have been studied extensively to identify the best style to increase workforce stability and nurses’ ITS. A systematic review by (Cowden et al., 2011), confirmed the positive relationship between transformational leadership, supportive work environments and staff nurses’ intent to remain in their current position, and they recommended that organisations should incorporate relational leadership theory into management practice to positively influence nurse retention. However, our findings demonstrate that it is the combination of supportive managers and supervisors as well as effective administrative processes and staff that collectively increase nurses’ ITS, rather than the use of a specific or single leadership style. Other studies support this (Hutchinson and Jackson, 2013; Sosik et al., 2011).

Another factor predicting nurses’ ITS was the level of organisational support. Empowerment along with organisation commitment and desire to stay accounted for the greatest variance of ITS in the study by Cowden and Cummings (2015). This underlines the importance of supportive managers enabling and empowering their staff. The overlap of ITS predictors identified in this review and organisation strategies should be recognised. For example, nursing empowerment can be achieved by providing nurses with opportunities for personal and professional development. Such opportunities could include the provision of preceptorship and/or mentorship, extending their education by providing a structured in-service education and supporting staff to apply for promotion (Brewer et al., 2016; Nedd, 2006; Ross, 2016). Initiatives for continuing professional development for nurses can influence nurses’ critical thinking abilities, improve their sense of control over their practice and strengthen their ITS (Yarbrough et al., 2017). Moreover, heavy workload, inadequate staffing, and increased nurses’ working hours were identified as factors that increased nurses’ job tension and decreased job satisfaction and ITS (Abernathy, 2007; Gilles et al., 2014; Liang et al., 2016).

Such retention and ITS initiatives require investment and resources. A theoretical proposition of a linear relationship between nurse satisfaction, quality of patient care, patient satisfaction, and nurse retention was provided by Newman and Maylor (2002). The main value of this theoretical framework is the understanding that the components are linked and interdependent. Each component requires attention if a supportive organisational climate is to be created (Sheopuri, 2019). Further evidence for this can be found if organisational classification based on Magnet accreditation is considered. Nurses who work in Magnet hospitals tend to remain employed longer than nurses working in non-Magnet institutions (Cram, 2013; Lacey et al., 2007). This is an outcome of such institutions having a supportive work environment which contributes to lower levels of nurse dissatisfaction and burnout (Kelly et al., 2012).

## Limitations of the review

Despite the added value of a systematic review approach, some limitations could impact its finding. Variability in the conceptualisations and measurement of work environment may limit the validity and generalisability of the findings because the dissimilar operational and theoretical definitions of constructs prevent direct comparison and so could lead to inconsistent findings. However, with these overlapping and complicated concepts, and regardless of the measures taken to ensure the search was comprehensive, such as piloting the search terms and librarian consultation, some relevant literature may have been missed.

A further limitation was associated with adopting the narrative synthesis approach to synthesise the evidence, which may have resulted in some bias and the generation of unsound conclusions. However, this approach provided an overarching framework, which ensured that the process was clear. Furthermore, using specific inclusion criteria and a well-structured search question, piloting proposed processes and use of independent reviewers mitigated potential bias. A high level of agreement between the reviewers was noted. The inclusion of quantitative studies published in English, may have led to exclusion of some potentially relevant research written in languages other than English. However, resources for translation were not available and so this was unavoidable. Similarly, “grey literature” was not included as identifying, locating, and retrieving the relevant material was beyond the scope of this review. Excluding the grey literature may therefore increase publication bias, reduce the reviews’ comprehensiveness and timeliness, and unbalance the picture of available evidence (Paez, 2017). However, some unpublished theses and dissertations were included because as this is a relatively new area of research interest emerging data can be found in recent doctoral studies which are available in university websites and repositories (Adams et al., 2016) which may have reduced the potential impact of publication bias.

## Conclusion

The workforce shortage in healthcare organisations is a global issue that will become more acute in the next 10 years (WHO, 2013). One way to meet this challenge is for organisations to retain more staff. Despite the limitations of this review, the evidence indicates that attention to meso-level variables, such as organisation characteristics and work environment, is vital to improve the working environment and to increase ITS. This review identified theoretical models of nurses’ ITS that have been developed and tested over time. These models were instrumental to differentiating ITS from other concepts such as ITL, turnover, and retention, both theoretically and operationally.

Although refinement of these models was achieved by including more variables and explaining direct and indirect relationships between the variables and ITS, further research is required to improve and enrich them. Personal individual characteristics are not easily modified, and the interaction between work environment elements and individual factors cannot be ignored. However, more attention should now be given to individual variables, such as organisational commitment and overall job satisfaction to learn more about their impact on ITS. Finally, the multifaceted nature of the concept of ITS makes it difficult to present definitive conclusions based on the findings of this review. However, elements of the work environment such as effective leadership practice, empowerment culture, professional development opportunities and a supportive organisation climate appear to be crucial factors for encouraging nurses to stay at their workplace.

**Key points for policy, practice and/or research**

• Concept of intention to stay is a multifaceted concept and requires more investigation in order to define the concept operationally and theoretically and differentiate it from similar concepts.
• The identified conceptual models of ITS were instrumental to differentiating this concept from other concepts such as intention to stay, turnover and retention.
• Evidence indicates that attention to meso-level variables, such as organisation characteristics and work environment, is vital to improve the working environment and to increase ITS.
• Personal individual characteristics are not easily modified, and the interaction between work environment elements and individual factors cannot be ignored. However, more attention should now be given to individual variables, such as organisational commitment and overall job satisfaction to learn more about their impact on ITS.
• Elements of the work environment such as effective leadership practice, empowerment culture, professional development opportunities, and a supportive organisation climate appear to be crucial factors for encouraging nurses to stay at their workplace.

## Supplemental Material

sj-pdf-1-jrn-10.1177_17449871221080731 – Supplemental Material for Nurses’ intention to stay in the work environment in acute healthcare: a systematic review Click here for additional data file. Supplemental Material, sj-pdf-1-jrn-10.1177_17449871221080731 for Nurses’ intention to stay in the work environment in acute healthcare: a systematic review by Asma Al Yahyaei, Alistair Hewisonor, Nikolaos Efstathiou, and Debbie Carrick-Sen in Journal of Research in Nursing
