# Supplementary material for: Nurses’ intention to stay in the work environment in acute healthcare: a systematic review
Source: J Res Nurs. 2022 Jul 8;27(4):374–97. doi: 10.1177/17449871221080731 (PMC9272506; doi:10.1177/17449871221080731)
Supplement: sj-pdf-1-jrn-10.1177_17449871221080731 – Supplemental Material for Nurses’ intention to stay in the work environment in acute healthcare: a systematic review [file sj-pdf-1-jrn-10.1177_17449871221080731.pdf]

Supplementary Table S 1:Summary table of the included studies

Author(s) Year/Country	Design	Sample and population/Setting/Response rate	Theoretical framework	Assessment tool	Reliability	Validity	Statistical analysis	Results and Quality appraisal score (QS)
Sourdif (2004)/Canada	A cross-sectional survey	108/221 drawn from a sample of 900 registered nurses working in a 400-bed university hospital in Montreal<br><br>RR: 49.90%	Organizational Dynamics Paradigm of Nurse Retention (Taunton et al., 1997)	Nurses' Intent to Stay Questionnaire (Taunton et al., 1997)	Cronbach's alpha ranged between 0.70 and 0.91	The tools had been used in previous studies. There was no confirmation of a formal validation study.	• Descriptive statistics<br>• ANOVA<br>• Pearson's correlation<br>• Linear regression	• 50.4% of the nurses reported that they have the intention to stay in their current job.<br>• 29.0% reported the intention to stay in their hospital.<br>• Only two variables were found to be statistically significant: satisfaction at work ($p = 0.022$) and satisfaction with administration ($p = 0.016$); they together explained 25.5% of the variance of intent to stay (F 2101 = 17.265; $p = 0.000$).<br><br>QS = 38 (high)
Nedd (2006)/ USA	A descriptive, correlation survey design	206 questionnaires were usable of the 275 returned questionnaires of a planned random sample of 500 from total 147,420 licensed nurses working in different health care settings in Florida, USA<br><br>RR: 42%	Kanter's Theory of Organizational Empowerment	• Job Activities Scale (JAS)<br>• Organizational Relationships Scale (ORS)<br>• Conditions for Work Effectiveness Questionnaire (CWEQ)<br>• 4 items assessed ITS developed by Kim, Price, Mueller and Watson (1996)	JAS: 0.81<br>ORS: 0.92<br>CWEQ: 0.96<br>4 items assessing ITS: 0.86	The tools had been used in previous studies. There was no confirmation of a formal validation study.	• Descriptive statistics<br>• Pearson correlation coefficients	• Nurses perceived moderate levels of empowerment at their workplaces.<br>• Intent to stay was significantly positively correlated with all empowerment variables.<br>• Individual characteristics, such as age, education and experience, were not significantly correlated with ITS.<br>• Tenure was not significantly related to intent to stay.<br><br>QS = 43 (high)
Tourangeau and Cranley (2006)/Canada	A descriptive survey design	8,456 nurses from 75 hospitals - registered nurses (RNs) and registered practical nurses working in acute-care hospitals in Ontario, Canada<br><br>RR: 65%	Determinants of Nurse Intention to Remain Employed based on the Conceptual Model of Intent to Stay (Boyle 1999)	• Ontario Nurse Survey, which includes the Maslach Burnout Inventory (MBI), the Revised Nursing Work Index (NWI-R) and the McCloskey Mueller Satisfaction Scale (MMSS)	MBI: 0.91<br>NWI-R: 0.84<br>MMSS: 0.56 or greater<br>ITS: not mentioned	The tools had been used in previous studies. There was no confirmation of a formal validation study.	• Descriptive statistics<br>• Multiple regression	• The regression models explained 34% of the variance in the nurses' intention to remain employed.<br>• The strongest predictors were nurse age, overall nurse job satisfaction and years of employment in the current hospital.<br>• Only four out of six proposed predictors were statistically significant determinants of nurse intention to remain: job satisfaction, personal characteristics of nurses, work group cohesion and collaboration and organizational commitment of nurses.<br>• The other two categories of predictors, nurse burnout and nurse manager ability and support, may be predictors of job satisfaction and have indirect effects on intention to remain employed that are mediated through job satisfaction.<br><br>QS = 43(high)

Abernathy (2007)/USA<br><br>PhD dissertation	A quantitative analysis approach using secondary data	581 staff nurses working in urban and suburban hospital settings<br><br>RR: Not mentioned	Herzberg Two-Factor Theory-Theoretical Framework	Secondary data collected by the Individual Workload Perception Scale – Revised	Cronbach's alpha ranged between 0.664 and 0.920.	The tools had been used in previous studies. There was no confirmation of a formal validation study.	• Descriptive statistics<br>• t-test<br>• Regression analyses	• The mean intent to stay score was 3.865 for those working in suburban settings and 3.731 for those working in an urban setting. The p value for the difference (0.134) was 0.0763, which was greater than 0.05 (no difference).<br>• Only satisfaction with workload was a significant predictor of intent to stay; however, peer support was marginally significant (p = 0.068, which is very close to 0.05). Both models had a good R-square explaining 52.88% and 34.09% of intent to stay, respectively.<br>• Higher peer support was positively associated with higher intent to stay in the linear regression model but not in the logistic regression model.<br><br>QS = 37 (high)
Cram (2013)/USA<br><br>PhD dissertation	A quantitative study utilizing the survey method	119/150 nurses working in inpatient acute care and ICUs in one magnet hospital and one non-magnet hospital in the southwestern United States<br><br>RR: Not mentioned	Represented by several leadership theories	Modified PES-NWI	The PES-NWI provided criterion-related validity and authenticates replications, which confirmed based on a study by Warshawsky and Havens (2011) that examined 20 previous studies utilizing PES-NWI.	The PES-NWI provided criterion-related validity and authenticates replications, which both correlate reliability, based on a study by Warshawsky and Havens (2011) that examined 20 previous studies utilizing PES-NWI.	• Descriptive statistics<br>• Independent-samples Mann-Whitney U test<br>• Chi-square test<br>• Related-samples McNemar's Test	• Significant differences existed between the magnet and non-magnet hospitals with regard to perceptions of leadership effectiveness and intention to remain with the organization.<br>• Nurses in the magnet hospital were more likely to rate leadership as more effective and were more likely to indicate their desire to remain with the hospital.<br>• There was a significant relationship between nursing leadership and work environment and nurses' intent to stay in the magnet status hospital versus the non-magnet status hospital.<br><br>QS = 36 (high)
Manning (2014)/USA<br><br>PhD dissertation	A descriptive, correlational design with a quantitative approach using survey instruments	441/500 staff nurses working in three acute-care hospitals<br><br>RR: 31%	• The Theoretical Model of Clinical Nurses Intent to Stay<br>• The Full Range Leadership Model<br>• Kanter's Theory of Structural Empowerment<br>• Schaufeli's Work Engagement Conceptual Framework	• Multifactor Leadership Questionnaire<br>• Conditions of Work Effectiveness Questionnaire<br>• Utrecht Work Engagement Scale<br>• Intent to Stay Questionnaire (Kim et al. (1996))	The validity and reliability of the tools had been established in previous studies ‹ITS: 0.89-0.90 Multifactor Leadership Questionnaire (MLQ): 0.76-0.89 CWEQ-II: 0.71-0.90 UWES: 0.72-0.84	The validity and reliability of the tools had been established in previous studies.	• Descriptive statistics<br>• Pearson Product Moment statistical analysis<br>• Multiple regression	• Transformational leadership style in nurse managers was not a significant predictor of staff nurse intent to stay in their current job or hospital for the next one, three or five years. None of the F values for these data sets had a p < 0.05.<br>• Transactional leadership style in nurse managers was not a significant predictor of staff nurse intent to stay in their current job or hospital for the next one, three or five years.<br>• Passive-avoidant leadership style was not a significant predictor of staff nurse intent to stay. No trends could be concluded from the multiple regression analysis.<br><br>QS = 42 (high)

Owens (2011)/USA<br><br>PhD dissertation	A single-stage design using a prospective, cross-sectional survey	328/1559 RNs and LVNs/LPNs employed by a large not-for-profit hospital system in Texas and nearby states<br><br>RR: Not mentioned	Self-Determination Theory by Deci and Ryan (1985)	• Teamwork and Safety Climate Survey (TSCS)<br>• 6-item intent to stay survey<br>• Nursing Work Index – Revised: Autonomy subscale (NWI-R)<br>• 4-item Job Satisfaction (JS) survey	• The overall reliability of the assessment tool ranged from 0.82-0.93	• All of the tools had been used previously in the literature except ITS and the Paths to Change scale.	• Descriptive statistics<br>• ANOVA<br>• Pearson correlation<br>• Multiple regression	• Supported previous studies by showing that satisfied nurses were more likely to stay. The JS scores were significantly correlated with intent to stay scores (r =.77, p <.001).<br>• Intent to stay was the only variable in which a significant difference between generational cohorts emerged in this study.<br><br>QS = 39 (high)
Riegal (2012)/USA<br><br>PhD dissertation	A quantitative, correlational approach using a cross-sectional survey design	231/1000 male and female registered, licensed nurses from North Carolina<br><br>RR: 21%	Social Exchange Theory (SET)	• Organizational Commitment Questionnaire<br>• Perceived Organizational Support Questionnaire<br>• 3 items assessed ITS	The reliability of the assessment tools had been established in previous studies.	A demographic survey had been piloted.<br><br>Other measures: The validity had been established in previous studies.	• Descriptive statistics<br>• Independent t-test<br>• ANOVA<br>• Linear regression	• The regression model was significant with 40% of the variance for thinking about leaving, 31.5% of the variance for looking for a new job and 30% of the variance for choosing to work again for the current organization explained by the eight predictor variables in the POSQ.<br>• POS aggregate analysis revealed an inverse relationship between thinking about leaving and looking for a new job. On the other hand, a positive predictive relationship was found between POS and choosing to work again for current organization.<br>• The POC aggregate analysis revealed an inverse relationship between thinking about leaving and looking for a new job and a positive predictive relationship between choosing to work again for current organization.<br><br>QS = 48 (high)
AbuAlRub et al. (2016)/Jorden	A descriptive correlational design	330/535 RNs and midwifes working in all health care centers and hospitals in two underserved governorates (Mafrq and Ma'an) in Jordan<br><br>RR: 61.68%	Not mentioned	• McCloskey Mueller Satisfaction Scale (MMSS)<br>• Revised Nurse Working Index (NWI-R)<br>• 1 item assessed ITS	MMSS: 0.95<br>NWI-R: 0. 93	The tools have been previously used and validated. The translated (Arabic) version was piloted.	• Descriptive statistics<br>• Pearson's correlation<br>• Logistic regression	• The mean overall job satisfaction was borderline (mean [SD] = 2.20 [.54]).<br>• The mean intention to stay at work was high (mean [SD] = 3.11 [.98]).<br>• Nurses perceived their work environment as unsupportive, with an overall mean of less than 2 (mean [SD] = 1.92 [.64]).<br>• The results showed a strong positive association between job satisfaction and work environment.<br>• The results of the logistic regression indicated that receiving housing, job satisfaction and work environment were the predicting variables of the level of intention to stay at work.<br><br>QS = 44 (high)

Al-Hamdan et al. (2017)/Jorden	A descriptive, cross-sectional survey design	650/1000 RNs working on inpatient units in three hospitals: one public and one private hospital in Amman and one teaching hospital in Irbid, Jordan<br><br>RR: 89.5%	Not mentioned	• Practice Environment Scale of the Nursing Work Index (PES-NWI)<br>• McCain Intent to Stay scale<br>• Quinn and Shepard's (1974) Global Job Satisfaction survey	PES-NWI Cronbach's alpha: 0.92<br><br>McCain's Cronbach's alpha: 0.74	Content validity of the Arabic version of the three instruments was established by experts in nursing. The instruments were translated and back translated by bilingual experts.	• Descriptive statistics<br>• Multivariate linear regression	• The nursing work environment was positively associated with the nurses' intent to stay (t = 4.83, p <.001).<br>• The Intent to Stay score increased by 3.6 points for every one-unit increase in the total PES-NWI score on average.<br>• The highest Intent to Stay scores were reported by nurses from public hospitals<br><br>QS = 44 (high)
Asamani et al. (2016)/Ghana	A quantitative approach using a cross-sectional survey design	273/275 staff nurses working in five primary and secondary hospitals in the eastern region of Ghana<br><br>RR: 99.30%	Guided by the Path-Goal Leadership theory	• Path-Goal Leadership Questionnaire (leadership style)<br>• 7-item job satisfaction scale<br>• 4 items assessed ITS	• Path-Goal Leadership style: 0.831<br>• Job satisfaction: 0.754<br>• ITS: 0.695	The validity of the tools had been established previously.	• Descriptive statistics<br>• Pearson correlation<br>• Regression	• The majority of nurses (51.7%) intended to leave their current workplace, and 20% were actively seeking opportunities to leave.<br>• There was a weak but significant positive correlation between supportive (r = 0.221), participative (r = 0.243) and achievement-oriented leadership styles (r = 0.184) and the staff's intentions to stay.<br>• The nurse managers' leadership styles statistically explained 13.3% of the staff's intention to stay at their current job position.<br><br>QS = 38 (high)
Brewer et al. (2016)/USA	A cross-sectional design that is part of a 10-year longitudinal panel design	Nurses who have been licensed for 7.5-8.5 years<br><br>RR: 69%	Expanded Price Model of Turnover (Price 2001)	• Transformational leadership scale<br>• 4 items assessed ITS<br>• OC: 6 items selected by Price (2001)<br>• 5 items assessed general job satisfaction	Cronbach's alpha ranged from 0.82 for job satisfaction to 0.95 for non-local job opportunity	Validity has been established through uses in multiple previous studies (Brewer & Kovner 2007, Kovner et al. 2007, 2009, 2014, Brewer et al. 2009, 2012).	• Descriptive statistics<br>• Ordered probit model	• Transformational leadership did not have a significant impact on intent to stay and job satisfaction but was significantly associated with organizational commitment.<br>• Organizational commitment, job satisfaction, mentor support, promotional opportunities and age were positively associated with intent to stay, whereas ethnicity, non-local job opportunities and work settings were negatively associated with intent to stay.<br><br>QS = 45 (high)

Cowden and Cummings (2015)/Canada	A correlational, mixed-method, nonexperimental design	Full-time and part-time RNs and LPNs who worked in acute-care study units in nine hospitals (two teaching hospitals and seven community hospitals)<br><br>RR: Not mentioned	Theoretical model of clinical nurses' ITS (Cowden & Cummings, 2012)	Quality Work Environment Study survey which includes:<br>• Leadership Scale (10 items; Estabrooks, Squires, Cummings, Birdsell, & Norton, 2009)<br>• Global Empowerment (2 items; Laschinger & Finegan, 2005)<br>• Areas of Work–Life Questionnaire (29 items; Leiter & Maslach, 1999)<br>• Maslach Burnout Inventory (9 items; Maslach, Jackson, & Leiter, 1996)<br>• Global Job Satisfaction (3 items; Quinn & Shephard, 1974)<br>• Stanford Safety Culture Instrument (16 items; Ginsberg, Norton, Casebeer, & Lewis, 2005)<br>• Revised Nursing Work Index Questionnaire (29 of 52 items; Aiken & Patrician, 2000)	• The Cronbach's alpha reliability coefficients for study items varied between.71 and.94.	• The tools have been used in previous studies.<br>• Survey questions were assessed for clarity, potential for confusion or misinterpretation, context and response options.	SEM	• The final model was estimated to explain 63% of the variance in ITS.<br>• Organizational commitment, empowerment and desire to stay were the model concepts with the strongest effects on nurses' ITS.<br>• Leadership practices indirectly influenced ITS.<br><br>QS = 40 (high)

El-Jardali et al. (2013)/Lebanon	A nonexperimental cross-sectional survey design	Registered nurses who were working in a total of 103 healthcare facilities (hospitals and PHC) in underserved areas in Lebanon<br><br>RR: 75.5%	Not mentioned	• McCloskey Mueller Satisfaction Scale<br>• NWI-R<br>• Career plans (ITS)	Reliability of the developed tool was not reported.	The content validity of the ITS tool had been evaluated.	• Descriptive statistics<br>• Student's sample t-test<br>• Logistic regression	• 35.1% of nurses indicated that they were likely or very likely to stay in their current job for the coming one to three years<br>• Surveyed nurses were most satisfied with their relationships with co-workers and least satisfied with extrinsic rewards.<br>• Rural nurses working in PHC centers were more satisfied than their hospital counterparts on all aspects of work and had a significantly higher intention to stay (62.5% compared with 31.5% in hospitals, $p < 0.001$).<br>• The regression analysis revealed that nurses less likely to report intent to stay were younger, unmarried, had fewer years of work experience and were not working toward a higher degree.<br>• The analysis revealed a directly proportional relationship between nurses' reported job satisfaction and their intent to stay.<br><br>QS = 41 (high)
Gilles et al. (2014)/Switzerland	A cross-sectional design	A total of 10,070 hospital employees including 9,108 who belonged to one of the following professional groups: physicians, nurses and care providers, laboratory staff, administrative staff, non-physician researchers, logistics staff (e.g., catering, cleaning personnel, technicians) and psycho-social staff. The remaining 962 employees were in apprenticeship, PhD students or had an external contract.<br>Nurses: 1,228<br><br>RR: 49.80%	The Conceptual Model of Intent to Stay (CMIS) adopted from Boyle et al. (1999)	• Copenhagen Burnout Inventory<br>• French Saphora Job Survey<br>• Modified Lausanne University Hospital survey<br>• A single item was used to measure job satisfaction and ITS	The Cronbach's alpha was calculated	The tools have been previously used and validated.	• Descriptive statistics<br>• Linear regression<br>• SEM	• For all professional categories, self-fulfillment increased intent to stay (all $\beta > 0.14$, $p < 0.05$).<br>• Burnout decreased intent to stay by weakening job satisfaction ($\beta < -0.23$ and $\beta > 0.22$, $p < 0.05$).<br>• Some factors were associated with specific professional categories: workload was associated with nurses' intent to stay ($\beta = -0.15$), and physicians' institutional identification mitigated the effect of burnout on intent to stay ($\beta = -0.15$ and $\beta = 0.19$).<br><br>QS = 40 (high)

Gregory et al. (2007)/Canada	A nonexperimental predictive survey design	458/1,173 RNs working in acute-care settings<br><br>RR: 29.40%	Based on the Conceptual Model of Behavioral Intentions (CMBI)	• The Employee Attitude Survey (EAS)<br>• Revised Impact of Health Care Reform Scale (RIHCRS)<br>• A modified version of the General Job Satisfaction Scale<br>• Organizational Commitment Questionnaire (Mowday, Steers, & Porter, 1979)<br>• The Intent to Stay scale was adapted from the Intent to Quit and Job Search Scales (Turnley & Feldman, 1998)	Emotional Climate: 0.82<br>Practice Issues: 0.76<br>Collaborative Relations: 0.86<br>Psychological Contract Violation: 0.70<br>General Job Satisfaction: 0.80<br>Organizational Commitment: 0.92<br>Intent to Stay: 0.72	The validity of the assessment tools had been established in previous and current studies using a factor analysis.	• Descriptive statistics<br>• SEM	• Despite moderate levels of job satisfaction, RNs held negative perceptions of culture (emotional climate, practice-related issues, and collaborative relations), trust and commitment and were unlikely to stay with their current employers.<br>• Based on the magnitude of their total effects, the determinants of intent to stay may be ranked as follows: satisfaction (B =.46), culture (B =.38) and trust (B =.25).<br>• Structural equation modeling provided support for the impact of culture, trust and satisfaction on commitment and partial support for intent to stay, explaining 45% and 31% of the variance, respectively.<br><br>QS = 40 (high)
Jiang et al. (2017)/China	A quantitative, cross-sectional and descriptive design	976/1137 registered nurses employed at the EDs of 78 secondary and tertiary hospitals in Shanghai<br><br>RR: 86.90%	Not mentioned	• Chinese version of the 'Maslach Burnout Inventory'<br>• One item assessed ITS and ITL	• Job satisfaction scale Cronbach's α: 0.94<br>• Burnout scale Cronbach's α ranged between 0.852 and 0.749	The tools had been piloted by a panel of six experts in the field of emergency nursing care.	• Descriptive statistics<br>• Non-paired t-test<br>• Analysis of variance (ANOVA)<br>• Multiple logistic regression	• Among the respondents, 75% reported being very satisfied or satisfied with their jobs, but there was a high level of burnout, and 22.5% of the nurses expressed their intention to leave the ED within the following year ($p < 0.05$).<br>• Nurses' satisfaction and burnout were associated with intention to leave.<br>• Salary, nurse–patient relationships, nurse staffing and work environment were areas where nurses were less satisfied, while group cohesion was associated with greater satisfaction.<br><br>QS = 44 (high)
Kaewboonchoo et al. (2014)/Thailand	Self - administered cross-sectional design	514/526 female registered nurses (RNs) working in two medium- and large-sized government hospitals in Phranakhon Si Ayutthaya Province, Thailand<br><br>RR: 97.70%	Not mentioned	• Thai version of the Job Content Questionnaire (JCQ)<br>• Four items measured ITS	• Cronbach's alpha coefficient for the JCQ scales and subscales ranged between 0.322 and 0.899<br>• Cronbach's alpha coefficient for intent to stay at work in this study was 0.861.	The tools had been used in the literature previously.	• Descriptive statics<br>• Independent t-test<br>• Multiple linear regression	• The prevalence of high job strain and low intent to stay at work were 17.5% and 22.4%, respectively.<br>• The mean (SD) scores of the nurses for psychological job demand, decision latitude, workplace social support and intent to stay at work were 33.5 (4.4), 70.7 (6.9), 23.8 (2.8) and 14.6 (2.9), respectively.<br>• The multiple regression analysis indicated that intent to stay at work was significantly correlated with only supervisor support among the nurses with high-strain jobs and with co-worker support in nurses with active jobs.<br><br>QS = 42 (high)

Lacey et al. (2007)/USA	A quantitative, cross-sectional and descriptive design, secondary analysis	3,337 RNs from 15 institutions, 11 geographically diverse states, and 292 nursing units from the repository number of nurses who had completed the Individual Workload Perception Scale (IWPS)<br><br>RR: Not mentioned	Maslow's Hierarchy of Needs.	The IWPS	IWPS Cronbach's alpha ranged from 0.61 to 0.91.	The tool validity had been established previously in the literature.	• Descriptive statistics<br>• ANOVA	• Nurses employed by organizations that have achieved magnet recognition have significantly more positive perceptions of support for nurses than do nurses employed by organizations that currently identify themselves as either magnet aspiring or non-magnet.<br>• All subscales of the IWPS were significantly better for magnet facilities (higher means are more positive), all with a p value of.000.<br>• Intent to stay had a statistically significantly higher mean score in magnet facilities (3.92) compared with aspiring magnet (3.72) and non-magnet (3.64) facilities.<br><br>QS = 30 (moderate)
Letvak and Buck (2008)/USA	A cross-sectional design	323 RNs employed in direct patient care at three hospitals in a southern U.S. state<br><br>RR:<br>First hospital, 13%<br>Second hospital, 39%<br>Third hospital, 31%	Not mentioned	• Four items assessed overall job satisfaction<br>• The Health Professions Stress Inventory (Wolfgang, 1988)<br>• Work Productivity and Activity Impairment Questionnaire:<br>• General Health (WPAI-GH)<br>• One item assessed ITS	The reliability of the instrument had been established previously by other studies. Cronbach's alpha coefficients of 0.85 and 0.90	The validity of the instrument had been established previously by other studies.	• Descriptive statistics<br>• Linear regression	• Assessment of intent to stay in hospital nursing indicated that 60% of participants plan on staying in nursing over the next five years, 25% were unsure and 15% plan on leaving.<br>• Predictor variables explained only 9% of the variance ($p < 0.001$) and included age (0.214), total years worked in nursing (0.204), quality of care provided (-0.142) and job satisfaction (-0.138).<br>• The most frequently reported reasons for leaving nursing for those who intended to leave were job stress (28.4%) and retirement (16.3%).<br><br>QS = 37 (high)
Liang et al. (2016)/Taiwan	A cross-sectional design	414/452 RNs working in hospitals in Taiwan<br><br>RR: 91.60%	Cowden and Cummings (2012) proposed a theoretical model of clinical nurses' ITS.	• The MLQ (Bass & Avolio 1997)<br>• The Safety Attitudes Questionnaire (SAQ) (Sexton et al. 2006)<br>• The Emotional Labor Questionnaire (ELQ) (Wu 2003)<br>• Four items assessed ITS (Wang et al. 2006)	MLQ: 0.96<br>SAQ: 0.89<br>ELQ: 0.89<br>ITS: 0.84	The tools have been previously used and validated.	• Descriptive statistics<br>• SEM	• The mean score for ITS was 3.19, indicating that the level of ITS appeared to be moderate for nurses. In total, 40.6% of nurses agreed or strongly agreed that they are willing to continue working in their current job.<br>• The proposed model explained 37% of the variance in ITS.<br>• ITS was positively correlated with age and the safety climate, whereas working hours per week and emotional labor were negatively correlated.<br>• The nursing position and transformational leadership indirectly affected intention to stay; this effect was mediated separately by emotional labor and the safety climate.<br><br>QS = 44 (high)

Mrayyan (2007)/Jorden	A descriptive comparative design	433/610 nurses working in teaching and non-teaching hospitals<br><br>RR: 71%	Not mentioned	MMSS, McCain Behavioral Commitment Scale (MBCS; McCloskey & McCain, 1987)	• MMSS Cronbach's alpha: 0.55-0.91<br>• MBCS Cronbach's alpha: 0.90	The tools have been used and validated previously.	• Descriptive statistics<br>• Chi-square test<br>• T-test<br>• Pearson product moment correlation	• Nurses in non-teaching hospitals intended to stay at their current jobs ($\bar{x}$ = 3.22, SD = 0.81) longer than nurses in teaching hospitals ($\bar{x}$ = 2.86, SD = 0.80; p =.01).<br>• The total score for nurses' job satisfaction was correlated with the total score of nurses' intent to stay (r =.452, p =.001), teaching hospitals (r =.231, p =.001) and non-teaching hospitals (r =.143, p =.001; Table 6). These correlations were significant at any level of.01.<br><br>QS = 36 (high)
Mrayyan (2008)/Jorden	A descriptive design using a survey method	362/550 Jordanian nurses who have been working at hospital settings for at least one year (12 hospitals; three governmental, three teaching and six private hospitals)<br><br>RR: 66%	Not mentioned	• Farly's Nursing Practice Environment Scale (NPES)<br>• McCain's Behavioral Commitment Scale (McCloskey, 1990)	• NPES Cronbach's alpha: 0.85<br>• MBCS Cronbach's alpha: 0.75	The validity of the tools had been established by previous studies.	• Descriptive statistics<br>• F-tests<br>• Stepwise regression	• Nurses in the entire sample reported that they plan to keep their jobs for at least two or three years, while the lowest rated mean was that nurses will not leave their present job under any circumstances.<br>• Hospitals' organizational climates, decision-making style, age and organizational structure were predictors of nurses' intent to stay.<br>• These four predictors explained 12% of the variance of nurses' intent to stay.<br><br>QS = 33(moderate)
Nakamura et al. (2010)/Japan	A cross-sectional survey design	319/370 full-time female nurses who work in three general hospitals operated by the same parent organization (JA Niigata Koseiren)<br><br>RR: 86.40%	Developed based on Management by Objectives (MBO)	• Developed survey<br>• One item assessed ITS	Not mentioned	Not mentioned	• Student's t-test<br>• Chi-square test<br>• Regression	• Five items were selected as significant independent variables on intention to remain employed by the stepwise multiple logistic regression analysis among 44 evaluation items for working conditions:<br>- Appreciating the hospital's atmosphere<br>- Utilizing ability<br>- Feeling an attachment to one's current job<br>- Consciousness of personal objectives<br>- Motivation<br><br>QS = 33(moderate)
Ross (2016)/USA	Online survey design	2,121 American Society of PeriAnesthesia Nurses members<br><br>RR: 22%	Not mentioned	The Individual Workload Perception Scale-Revised (IWPS-R)	Cronbach's alpha ranged between 0.68 and 0.92.	The tool validity had been established previously in the literature.	• Descriptive statistics<br>• Independent t-test<br>• ANOVA<br>• Tukey's post-hoc test	• Those with an assigned preceptor had a statistically significant higher ITS score (4.12) compared with those with no assigned preceptor (3.97).<br>• Perianesthesia nurses who had an assigned preceptor had significantly higher perceptions of organizational support (manager, peer and unit), workload, intent to stay and overall nursing satisfaction.<br>• Certified nurses have more positive perceptions of workload (p =.00) and intent to stay (p =.00) than noncertified nurses.<br><br>QS = 44(high)

Wang et al. (2017)/China	A cross-sectional descriptive design	535/623 RNs from four general hospitals in Shanghai, China<br><br>RR: 85.9%	Developed based on a literature review	• Wong and Law Emotional Intelligence Scale (Law et al., 2004)<br>• Chinese version of the transformational leadership scale (Li & Shi, 2005)<br>• Six items assessed ITS (Tao and Wang, 2010)	EI scale Cronbach's alpha: 0.86<br>TL scale Cronbach's alpha: 0.90<br>ITS Cronbach's alpha: 0.79	The validity of the tools has been established by previous studies.	• Descriptive statistics<br>• SEM<br>• Maximum likelihood estimation method	• The score of intent to stay ranged from 1.67 to 5, with a mean of 3.85 and an SD of 0.82.<br>• Transformational leadership had an indirect effect on nurse intent to stay through the mediator of nurse emotional intelligence; the mediator value was.111 (.663*.168 =.111).<br>• Therefore, the total effect of transformational leadership on nurse intent to stay was.486 (.375 +.111 =.486), and the indirect effect comprised 22.8% of the total.<br>• Overall, transformational leadership and nurse emotional intelligence accounted for 34.3% of the variance in nurses' intent to stay.<br><br>QS = 37(high)
Wang et al. (2012)/China	A cross-sectional survey	919/1120 nurses who had worked full-time for at least 1 year at the targeted hospitals<br><br>RR: 82%	Based on Tourangeau & Cranley's (2006) 'theoretical model of determinants of nurse intention to remain employed'.	• Chinese Nurses Job Satisfaction Scale (NJSS) (Tao et al. 2009)<br>• Chinese version of the OCS (Pei 2007)<br>• Chinese version of the Nurses' Intent to Stay (ITS) scale (Tao & Wang 2010)	• OCS Cronbach's alpha: 0.784 to 0.812<br>• NJSS Cronbach's alpha: 0.713 to 0.801<br>• ITS Cronbach's alpha: 0.792	The validity of the tools had been established in a previous study	• Descriptive statistics<br>• Spearman's correlation<br>• Independent sample t-test<br>• A stepwise regression	• Regression models explained 41.3% of the variance in nurses' ITS.<br>• Although the proposed model hypothesized 18 predictors of ITS, only seven were statistically significant factors of nurses' ITS: normative commitment, economic costs commitment, age, limited alternatives commitment, praise/recognition, professional advancement opportunities and the hospital classification.<br>• Of these, the strongest predictors were normative commitment, economic costs commitment and age, which explained 37.3% of variance in nurses' ITS.<br><br>QS = 42 (high)
Ke and Hung (2017)/Taiwan	A cross-sectional design	207/214 RNs working in two medical centers and two regional teaching hospitals in southern Taiwan. Hospital A: 48 nursing units and 1,200 nurses. Hospital B: 36 units and 1,175 nurses. Hospital C (teaching unit): 30 units and 687 nurses.<br>Hospital D: 18 units and 361 nurses.<br><br>RR: 100%	Developed based on a literature review	• Three-item Nurses' Intent to Stay Scale (NISS)<br>• The Organizational Climate Scale was modified from the scale developed by Litwin and Stringer (1968)	The reliability of the tool had been established, NISS: 0.75	NISS - content validity was assessed with eight experts who modified the tool; content validity index value = 1.00	• Descriptive statistics<br>• ANOVA<br>• Stepwise multiple regression	• Significant differences in nurses' ITS at their hospitals by working unit ($F(4, 202) = 4.09$, $p = 0.00$).<br>• Nurses working in special units had a higher ITS at their hospitals than did those who worked in ICUs.<br>• There was a significant positive correlation ($r = 0.19$, $p = 0.00$) between hospital OC rating and nurses' ITS at their hospitals. A higher hospital organizational climate was associated with a stronger ITS at the current hospital.<br>• Age, personal choice of working in the current hospital, working in an ICU and OC were predictors of nurses' ITS; these factors accounted for 50.8% of the total variance.<br><br>QS = 42 (high)

Yarbrough et al. (2017)/USA	A descriptive correlational study, mixed	RNs (n = 67) from a mid-sized hospital in a metropolitan area in the Southwestern United States<br><br>RR: 56%	Based on Fishbein and Ajzen's Expectancy–Value Theory	• A single item assessed ITS<br>• The Nurses Professional Values Scale-Revised (NPVS-R)<br>• Nurse Job Satisfaction Index<br>• Perceived Development Climate	• NPVS-R Cronbach's alpha: 0.94<br>• NJSI Cronbach's alpha: 0.76<br>• PDC Cronbach's alpha: 0.91	NPVS-R: 4 judges reviewed and confirmed content validity<br>•NJSI not described but used in a previous study<br>•PDC not described but used in a previous study	• Descriptive statistics<br>• Correlation statistics	• Neither professional values orientation nor its component subscales were significantly correlated with ITS.<br>• There was a strong correlation between professional values and career development ($r = 0.39$, $p < 0.001$).<br>• Both job satisfaction ($r = 0.39$, $p = 0.003$) and career development ($r = 0.29$, $p = 4$ 0.037) were positively correlated with retention.<br><br>QS = 37 (high)

Data Extraction Form adapted from the Cochrane Collaboration

Title of the systematic review: **Nurses' intention to stay in the work environment in acute healthcare**

This form has been established by adopting and modifying the "Data collection form for intervention review – RCTs and non-RCTs" of The Cochrane Collaboration. Particular new sections have been added into this tool and the inappropriate sections have been removed from the original form. Information included on this form should be comprehensive and may be used in the text of the review.

Notes on using this data extraction form:

- Be consistent in the order and style you use to describe the information for each included study
- Record any missing information as unclear or not described, to make it clear that the information was not found in the study report(s), not that you forgot to extract it.
- Include any instructions and decision rules on the data collection form, or in an accompanying document. It is important to practice using the form and give training to any other authors using the form.
- You will need to protect the document in order to use the form fields (Tools / Protect document)

Review title or ID	
Study ID *(surname of first author and year first full report of study was published e.g. Smith 2001)*	
Notes	

General Information

Date form completed *(dd/mm/yyyy)*	
Name/ID of person extracting data	
Reference citation (Doi #)	If available
Publication type (*e.g. full report, abstract, letter)*	☐ *Journal article.* ☐ *Thesis/ Dissertation.* ☐ *SR*
Notes:	

Study eligibility

Study Characteristics	Eligibility criteria *(Insert inclusion criteria for each characteristic as defined in the Protocol)*	Eligibility criteria met? Yes	No	Unclear	Location in text or source *(pg & ¶/fig/table/other)*
Type of study (as described in the paper)		☐	☐	☐	
Participants		☐	☐	☐	

Setting		☐ ☐ ☐	
The healthy work environment attributes	•	☐ ☐ ☐	
Types of outcome measures (ITS or ITL)	•	☐ ☐ ☐	
INCLUDE ☐	EXCLUDE ☐		
Reason for exclusion			
Notes:			

DO NOT PROCEED IF STUDY EXCLUDED FROM REVIEW

Characteristics of included studies

Methods

	Descriptions as stated in report/paper		Location in text or source *(pg & ¶/fig/table/other)*
**Aim of study** *(e.g. efficacy, equivalence, pragmatic)*			
**Dependent variables have been studied**			
**Independent variables have been studied**			
**Design** *(e.g. parallel, crossover, non-RCT)*			
**Ethical approval needed/ obtained for study**	☐ Yes ☐ No ☐ Unclear		
**Notes:**			

Participants

	Description *Include comparative information for each intervention or comparison group if available*		Location in text or source *(pg & ¶/fig/table/other)*
**Population description** *(from which study participants are drawn)*			
Setting *(including location and social context)*			
Inclusion criteria			
Exclusion criteria			
Method of recruitment of participants *(e.g. phone, mail, clinic patients)*			
Informed consent obtained	☐ Yes ☐ No ☐ Unclear		
Sample size			
Sample size justification, or calculation	☐ Yes ☐ No ☐ Unclear		
Response rate			
Subgroup analysis	☐ Yes ☐ No ☐ Unclear		

Notes:

Outcomes.

Outcome

	Description as stated in report/paper	Location in text or source *(pg & ¶/fig/table/other)*
Outcome name		
Outcome definition *(with diagnostic criteria if relevant)*		

Person measuring/ reporting			
Is outcome/tool validated?	☐ Yes ☐ No ☐ Unclear		
Management of missing data *(e.g. assumptions made for ITT analysis)*			
the reliability and validity have been evaluated			
Power *(e.g. power & sample size calculation, level of power achieved)*			
Notes:			

Other

**Study funding sources** *(including role of funders)*	☐ Yes ☐ No ☐ Unclear	
**Possible conflicts of interest** *(for study authors)*	☐ Yes ☐ No ☐ Unclear	

**Notes:**

Data analysis and result

	Description as stated in report/paper	Location in text or source *(pg & ¶/fig/table/other)*
The theoretical Framework		
Results (main result about the relationship between the healthy work environment and ITS or ITL)		
Any other results reported		
No. missing data		
level of analysis *(by individuals, unit, hospital)*		
Statistical test used and appropriateness to answer the research's questions*(e.g. adjustment for correlation)*	•	
Reanalysis required? *(specify, e.g. correlation adjustment)*	☐ Yes ☐ No ☐ Uncertain	

Reanalysis possible?	☐ Yes ☐ No ☐ Uncertain		
Notes:			

Discussion

	Description as stated in report/paper	Location in text or source *(pg & ¶/fig/table/other)*

**Key discussion points** *(with references in detail*		
Notes:		

limitations

	Description as stated in report/paper	Location in text or source *(pg & ¶/fig/table/other)*
**Limitation**		
**Strategies to overcome the limitations**	☐ Yes ☐ No ☐ Unclear	
Notes:		

Recommendations

	Description as stated in report/paper	Location in text or source *(pg & ¶/fig/table/other)*
Intended audience (State)		
Recommendations for research	☐ Yes ☐ No ☐ Unclear	
If “Yes” state them (briefly)		
Notes:		

Other information

	Description as stated in report/paper	Location in text or source *(pg & ¶/fig/table/other)*
**Key conclusions**		
**References to other relevant studies**		
**Correspondence required for further study information** *(from whom, what and when)*	☐ Yes ☐ No ☐ Maybe	
**Notes:**		

Study Quality Assessment

		Yes (3)	NO (0)	Partially (2)	Not clear (1)
1	The aim is clear				
2	Study design is appropriate doe the identified aim?				
3	The sample size was justified				
4	The target population is clearly defined				
5	The sample selection process was clear and representative for the target population				
6	The informed consent was obtained prior to data collection				
7	Measures has been undertaken to deal with non-responders and missing data				
8	The study instrument and measurement were piloted, trailed or published previously				
9	The statistical significance or precision estimate are clear(p value or confidence interval)				
10	The method including the statistical test included and clearly described				
11	The analysis of the basic data was described				
12	The response rate was mentioned and justified				
13	The result was presented in order to answer the study question(s)				

**14**	**The study conclusion and discussion were justified by the result**				
**15**	**The limitations of the study were discussed**				
**16**	**There were no conflict of interest or funding resources which may affect the study result**				
**17**	**The ethical approval was obtained**				
	**Total**				

Circle the appropriate quality level:

**High quality paper**	**51-34**
**Medium quality paper**	**33-17**
**Low quality paper**	**16-0**

Final decision	
To be included	
To be excluded	
